# Phenotypic and Genetic Predictors of Pathogenicity and Virulence in *Flavobacterium psychrophilum*

**DOI:** 10.3389/fmicb.2019.01711

**Published:** 2019-07-24

**Authors:** Krister Sundell, Lotta Landor, Pierre Nicolas, Jóhanna Jørgensen, Daniel Castillo, Mathias Middelboe, Inger Dalsgaard, Valentina Laura Donati, Lone Madsen, Tom Wiklund

**Affiliations:** ^1^Laboratory of Aquatic Pathobiology, Environmental and Marine Biology, Åbo Akademi University, Turku, Finland; ^2^Unité Mathématiques et Informatique Appliquées du Génome à l’Environnement (MaIAGE), Institut National de la Recherche Agronomique, Université Paris-Saclay, Jouy-en-Josas, France; ^3^Marine Biological Section, Department of Biology, University of Copenhagen, Helsingør, Denmark; ^4^Unit for Fish and Shellfish Diseases, National Institute of Aquatic Resources, Technical University of Denmark, Kongens Lyngby, Denmark

**Keywords:** *Flavobacterium psychrophilum*, bacterial cold-water disease, rainbow trout (*Oncorhynchus mykiss*), multilocus sequence typing (MLST), bacteriophage, LD_50_, pathogenicity, virulence

## Abstract

*Flavobacterium psychrophilum* causes bacterial cold-water disease (BCWD) in farmed rainbow trout (*Oncorhynchus mykiss*), with the multilocus sequence typing (MLST) clonal complex (CC) CC-ST10 accounting for the majority of outbreaks globally. The development of alternative strategies to antibiotic treatment of BCWD using bacteriophage-based control of *F. psychrophilum*, or virulence factors as targets for therapy, requires knowledge of the phage-sensitivity of outbreak strains and of universal traits contributing to their pathogenicity. To examine the association between virulence and both genetic (MLST sequence type (ST) and PCR-serotype) and phenotypic characteristics (adherence, antibiotic resistance, colony spreading motility, hemolytic and proteolytic activity), the median lethal dose (LD_50_) of 26 geographically disparate *F. psychrophilum* isolates was determined in rainbow trout. Furthermore, the *in vitro* sensitivity of the isolates against five bacteriophages was determined by the efficiency of plating (EOP). The tested *F. psychrophilum* isolates were mainly represented by CC-ST10 genotypes (22 out of 26) and showed up to 3-log differences in LD_50_ (8.9 × 10^3^ to 3.1 × 10^6^ CFU). No association between MLST ST and virulence was found because of a high variation in LD_50_ within STs. All identified serotypes (0, 1, and 2) were pathogenic, but ten most virulent isolates belonged to serotype 1 or 2. Isolates of high (LD_50_ < 10^5^ CFU), moderate (LD_50_ = 10^5^–10^6^ CFU), and weak (LD_50_ > 10^6^ CFU) virulence were similar in phenotypic characteristics *in vitro*. However, the only non-virulent CC-ST10 isolate was deficient in spreading motility and proteolytic activity, indicating that the characteristics are required for pathogenicity in *F. psychrophilum*. Univariate correlation studies found only non-significant associations between LD_50_ and the measured phenotypic characteristics, and the multivariable analysis did neither reveal any significant predictors of virulence. The majority of isolates (16 out of 26) were sensitive to at least four bacteriophages, with up to a 6-log variation in the EOP. Most CC-ST10 isolates (16 out of 22) were sensitive to the examined phages, including 5 out of the 7 most virulent isolates represented by prevalent and antibiotic-resistant STs. Our findings suggest that control of BCWD using lytic phages or interventions targeting shared characteristics of pathogenic *F. psychrophilum* strains should be further explored.

## Introduction

*Flavobacterium psychrophilum* is the etiological agent of Bacterial Cold-Water Disease (BCWD) causing devastating losses to farmed salmonid fish particularly during early freshwater growth stages. In the Baltic Sea region in Northern Europe, the pathogen has also been isolated several times from diseased juvenile salmonid fish in brackish water fish farms (Madetoja et al., 2002; Nilsen et al., 2011) and from brood stock carriers returning from the sea to spawn (Ekman et al., 1999). However, the largest problems caused by *F. psychrophilum* are still associated with early life stages of rainbow trout (*Oncorhynchus mykiss*) fry in hatcheries where Rainbow Trout Fry Syndrome (RTFS), here used interchangeably with BCWD, can result in high mortalities if left untreated. Today BCWD outbreaks in farmed fish are controlled by antibiotics administered through feed, which may lead to the dispersal of antibiotics in the environment. The lack of efficient preventative measures and obstacles in developing protective vaccination strategies against *F. psychrophilum* (Gomez et al., 2014) may in part be due to the genetic and serological diversity within the species and the insufficient knowledge of specific virulence factors. Virulence factors aid pathogenic bacteria to adhere to and invade the host, cause disease, and to evade host defense mechanisms. To be able to design alternative control strategies that inhibit the bacterium from causing disease, understanding which bacterial characteristics contribute to virulence in a susceptible host is of crucial importance.

Several putative virulence genes have been identified in genome analyses of *F. psychrophilum* (Duchaud et al., 2007, 2018; Castillo et al., 2016), but the role played by different factors in its pathogenicity in rainbow trout is still poorly understood. Attempts to link serologic (Lorenzen and Olesen, 1997; Dalsgaard and Madsen, 2000) and phenotypic characteristics, such as adhesion to host tissue (Nematollahi et al., 2003), hemolytic activity (Högfors-Rönnholm and Wiklund, 2010a) and protease production (Bertolini et al., 1994; Dalsgaard and Madsen, 2000; Ostland et al., 2000) to *F. psychrophilum* virulence have shown varying results. Studies with *F. psychrophilum* mutants have shown a connection between attenuated virulence and deficiency in motility and secretion of proteases and adhesins (Pérez-Pascual et al., 2015, 2017). However, such studies usually involve evaluation of a single or a few virulence factors at a time, undermining the fact that virulence could be the result of their combined effects. To date, only a few studies (Rangdale, 1995; Garcia et al., 2000; Madsen and Dalsgaard, 2000; Madetoja et al., 2002; Jarau et al., 2018) have evaluated the virulence of several *F. psychrophilum* isolates in rainbow trout, at least in part due to the lack of a challenge model that mimics natural disease. Waterborne infection models with *F. psychrophilum* have met with limited success in consistently inducing mortality in rainbow trout unless scarification (Madsen and Dalsgaard, 1999; Madetoja et al., 2000; Long et al., 2014) or a susceptible isogenic rainbow trout line (Pérez-Pascual et al., 2017) have been used. However, strain-dependent mortality rates can reproducibly be induced when *F. psychrophilum* is injected subcutaneously (Madetoja et al., 2002), intraperitoneally (Rangdale, 1995; Madsen and Dalsgaard, 1999, 2000; Jarau et al., 2018) or intramuscularly (Garcia et al., 2000; Madetoja et al., 2002) into rainbow trout. Despite the documented variation in virulence between isolates, reliable predictors of *F. psychrophilum* virulence have not yet been identified.

Individual *F. psychrophilum* isolates also vary in susceptibility to natural killers of bacteria, the bacteriophages (Stenholm et al., 2008). Bacteriophages with a strong lytic potential against *F. psychrophilum* have been discovered (Stenholm et al., 2008; Castillo et al., 2012) and phage-therapy has been proposed as an alternative strategy to antibiotic treatment for use in fish farms. A reemerging challenge for the use of bacteriophages to prevent or control *F. psychrophilum* in fish farm environments is the high degree of diversity found both within infective populations of the pathogen (Sundell et al., 2013) and phages (Stenholm et al., 2008; Castillo and Middelboe, 2016). For phage-therapy to be useful in aquaculture applications, more information is needed concerning the efficiency of phages against recurring virulent variants of *F. psychrophilum*.

Several epidemiological studies using a standardized multilocus sequence typing (MLST) approach for genetic typing of *F. psychrophilum* from wide geographical areas have proposed an epidemic population structure (Nicolas et al., 2008; Siekoula-Nguedia et al., 2012; Avendaño-Herrera et al., 2014; Nilsen et al., 2014), whereby one or several successful genotypes clonally expand and diversify overshadowing the underlying pathogen population. These epidemic clones have been suggested to represent highly adapted host-specific strains potentially more virulent compared to endemic or environmental strains (Siekoula-Nguedia et al., 2012). In a large-scale epidemiological study done by Nilsen et al. (2014) in countries around the Baltic Sea, the overwhelming majority of BCWD outbreaks in rainbow trout since the 1980s were attributed to a single clonal complex (CC), CC-ST10, comprising several closely related genotypes. Interestingly, CC-ST10 is also the predominant CC in several other European countries (Siekoula-Nguedia et al., 2012; Strepparava et al., 2013) as well as in Chile (Avendaño-Herrera et al., 2014) and in the United States (Van Vliet et al., 2016; Knupp et al., 2019) indicating a global distribution. Severe BCWD outbreaks have been associated with genotypes of CC-ST10 (Nilsen et al., 2014; Van Vliet et al., 2016) whereby virulence has been considered as a major contributor to its dominance in rainbow trout farms over both time and space. It has also been proposed that a higher ability to resist antimicrobial agents and to adhere to inert and fish surfaces are factors contributing to the persistence of CC-ST10 genotypes in fish farm environments (Sundell and Wiklund, 2015; Duchaud et al., 2018). Recent evidence also shows that virulent antibiotic-resistant strains of CC-ST10 have already replaced sensitive variants in some farms in the Baltic Sea region (Söderlund et al., 2018), calling for urgent development of alternative BCWD control strategies.

The epidemiological data collected in the large scale *F. psychrophilum* MLST studies (Nilsen et al., 2014; Knupp et al., 2019) indicate that development of efficient control strategies against BCWD in rainbow trout should be directed toward genotypes of CC-ST10 or target specific virulence factors expressed by them. Targeting CC-ST10 using lytic bacteriophages isolated from the environment is theoretically an ideal approach, but so far, limited information is available regarding the phage-susceptibility of current virulent outbreak strains of *F. psychrophilum* in the Baltic Sea region. To identify current virulent strains of *F. psychrophilum*, with special focus on CC-ST10, we used a reproducible intramuscular injection-based *in vivo* challenge model for rainbow trout to obtain a LD_50_ measure of relative virulence for current and past outbreak-related isolates from distinct geographical locations. Based on the obtained LD_50_ values, we assessed the potential of genetic (MLST profile and serotype) and phenotypic factors (adherence, antimicrobial resistance, colony morphology, hemolytic activity, motility and proteolytic activity) as predictors of, or contributors to, *F. psychrophilum* virulence. In addition, we inquired the potential of bacteriophages as therapeutic vehicles against BCWD by examining the *in vitro* phage susceptibility of highly virulent CC-ST10 genotypes.

## Materials and Methods

### Bacterial Isolation and Culture Preparation

Twenty-five isolates of *F. psychrophilum* included in this study were obtained from visceral organs of farmed rainbow trout in association with BCWD outbreaks in the Baltic Sea region in northern Europe between 1995 and 2017 (Table 1). For comparative analyses, the type strain NCIMB1947^T^ isolated from coho salmon (*O. kisutsch*), previously shown to be weakly virulent in rainbow trout (Madsen and Dalsgaard, 2000), was included as a reference strain. The isolates were cultured on tryptone yeast extract salts (TYES) agar (Holt et al., 1993), consisting of 0.4% tryptone, 0.04% yeast extract, 0.05% CaCl_2_ × 2H_2_O, 0.05% MgSO_4_ × 7H_2_O and 1.1% agar (pH 7.2) on which rough or smooth colony morphology (Högfors-Rönnholm and Wiklund, 2010b) was determined using a stereomicroscope. For species identification, the *F. psychrophilum*-specific primers PSY1 and PSY2 (Toyama et al., 1994) were used for PCR amplification of a partial fragment (1089 bp) of the 16S rRNA gene followed by electrophoretic confirmation of the amplification product. For test culture preparation, cryopreserved (−80°C) stock cultures of each *F. psychrophilum* isolate were cultured for 5 days on TYES agar at 15°C, followed by subcultivation in TYES broth for 48 h at 15°C under 200 rpm agitation. The bacterial cells of each broth culture were washed in fresh cooled TYES broth by centrifugation (5310 × *g*, 15 min, 4°C) and the optical density (OD) was spectrophotometrically adjusted to 1.0 at 520 nm, corresponding to a *F. psychrophilum* concentration of approximately 10^9^ CFU mL^–1^. The 48 h cultures were used for all challenge experiments and phenotypic characterization studies unless otherwise noted.

**TABLE 1:** The virulence of 26 *Flavobacterium psychrophilum* isolates listed by the median lethal dose (LD_50_) value in juvenile rainbow trout.

**Isolate**	**Host species**	**Virulence**	**LD_50_ (CFU)**	**ST**	**CC**	**Serotype**	**Country**	**Year**
FPS-G1	Rainbow trout	High	8.90 × 10^3^	89^*^	10	1	Germany	2017
FPS-F15	Rainbow trout	High	2.22 × 10^4^	2^*^	10	1	Finland	2017
FPS-P1	Rainbow trout	High	5.93 × 10^4^	2^*^	10	1	Poland	2016
FPS-R9	Rainbow trout	High	7.33 × 10^4^	329^*^	1	1	Russia	2017
FPS-P3	Rainbow trout	High	7.60 × 10^4^	2^*^	10	1	Poland	2017
FPS-S6	Rainbow trout	High	8.24 × 10^4^	92^*^	10	2	Sweden	2017
160401-1/5N	Rainbow trout	High	9.83 × 10^4^	92^*^	10	2	Denmark	2016
P30-2B/09	Rainbow trout	Moderate	1.37 × 10^5^	91	10	2	Finland	2009
F164	Rainbow trout	Moderate	1.40 × 10^5^	240	10	2	Sweden	1996
FPS-F16	Rainbow trout	Moderate	1.44 × 10^5^	2^*^	10	1	Finland	2017
V46	Rainbow trout	Moderate	1.50 × 10^5^	138	138	0	Finland	2005
FPS-S11A	Rainbow trout	Moderate	1.85 × 10^5^	92^*^	10	2	Sweden	2017
990512-1/2A	Rainbow trout	Moderate	2.06 × 10^5^	2	10	2	Denmark	1999
141127-1/2N	Rainbow trout	Moderate	3.56 × 10^5^	92^*^	10	2	Denmark	2014
P15-8B/11	Rainbow trout	Moderate	3.58 × 10^5^	2	10	1	Finland	2011
160401-1/5M	Rainbow trout	Moderate	3.73 × 10^5^	92^*^	10	2	Denmark	2016
FPS-F27	Rainbow trout	Moderate	4.33 × 10^5^	2^*^	10	1	Finland	2017
FPS-R7	Rainbow trout	Moderate	6.16 × 10^5^	328^*^	S	0	Russia	2017
FPS-S9	Rainbow trout	Moderate	6.83 × 10^5^	2^*^	10	1	Sweden	2017
030522-1/1	Rainbow trout	Moderate	7.57 × 10^5^	2	10	2	Denmark	2003
FPS-S10	Rainbow trout	Weak	1.36 × 10^6^	2^*^	10	1	Sweden	2017
010418-2/1	Rainbow trout	Weak	2.13 × 10^6^	10	10	2	Denmark	2001
K9/00	Rainbow trout	Weak	2.62 × 10^6^	79	10	1	Finland	2000
950106-1/1	Rainbow trout	Weak	2.64 × 10^6^	2	10	1	Denmark	1995
NCIMB1947^T^	Coho salmon	Weak	3.10 × 10^6^	13	9	0	United States	Unknown
FPS-S11B	Rainbow trout	Non-virulent	ND	92^*^	10	2	Sweden	2017

The virulence is expressed as high (LD_50_ < 10^5^), moderate (LD_*50*_ = 10^5^–10^6^), or weak (LD_50_ > 10^6^). The non-virulent isolate FPS-S11B was excluded from further statistical analysis. The sequence type (ST) was assigned after MLST in present^*^ and previous studies (pubmlst.org/fpsychrophilum). The clonal complex (CC) was named according to the predicted ancestral ST in an eBURSTv3 analysis (eburst.mlst.net). CC, clonal complex; CFU, colony forming units; LD_50_, median lethal dose; ND, not defined; S, singleton not belonging to a clonal complex; ST, sequence type.

### Genetic Characterization of *F. psychrophilum*

#### Multilocus Sequence Typing and Data Analysis

Extraction and purification of genomic DNA of each *F. psychrophilum* isolate for MLST was done using the NucleoSpin^®^ Microbial DNA kit (Macherey-Nagel) according to the manufacturer’s instructions. For MLST, partial sequences from *atpA*, *dnaK*, *fumC*, *gyrB*, *murG*, *tuf*, and *trpB* genes of the 16 previously untyped isolates (Table 1) were amplified by PCR according to the protocol described by Siekoula-Nguedia et al. (2012). Both strands of the amplified products were Sanger sequenced using the corresponding forward and reverse PCR primers. All chromatograms were manually checked for quality before assignation of the allele type (AT) and sequence type (ST) using an in-house script (P. Nicolas, INRA). Reference strains were included as internal controls for the MLST procedure and the assigned ATs and STs were submitted to the *F. psychrophilum* MLST database^1^. Following the assignation of ATs and STs, CCs were identified using eBURSTv3^2^. The predicted ancestral genotype (founder) of a CC was defined as the ST with the highest number of single locus variants. STs not belonging to any CC were referred to as singletons.

#### PCR Serotyping

For serotyping of all isolates used in this study, a multiplex PCR-based serotyping scheme for *F. psychrophilum* developed by Rochat et al. (2017) was used. The multiplex PCR reactions were performed using 5 μL purified genomic *F. psychrophilum* DNA (NucleoSpin^®^ Microbial DNA, Macherey-Nagel) as the template in a 50 μL reaction mixture consisting of 2 units of DyNAzyme II DNA polymerase, 1× Optimized DyNAzyme buffer (Thermo Fisher Scientific), 10 mM dNTPs together with 10 μM of the four primer pairs described by Rochat et al. (2017). The PCR reaction mixture was heated at 94°C for 2 min, followed by 30 cycles of 94°C for 30 s, 52°C for 30 s, 72°C for 60 s, and a final extension at 72°C for 10 min. The amplified PCR products were electrophoresed (4 V cm^–1^, 60 min) on a 1% agarose-Tris-borate-EDTA gel stained with ethidium bromide and visualized under ultraviolet transillumination (Alpha Innotech Multi Image Light Cabinet). A 100 bp DNA ladder was used for fragment size estimation and subsequent classification into type 0 (188 bp), 1 (188 and 549 bp), 2 (188 and 841 bp), or 3 (188 and 361 bp).

### Phenotypic Characterization of *F. psychrophilum*

#### Antibiotic Susceptibility Testing

The antibiotic susceptibility of the *F. psychrophilum* isolates was tested through the Kirby-Bauer disk diffusion method on diluted (1:5) Mueller-Hinton agar medium supplemented with 5% w/v fetal calf serum. For each isolate, a 40-μL volume of bacterial TYES broth suspension containing approximately 10^9^ CFU mL^–1^ of *F. psychrophilum* was transferred into 5 mL phosphate-buffered saline (PBS) and poured onto the agar plates. After removing excess suspension, antibiotic disks (florfenicol 30 μg, oxolinic acid 2 μg, sulfamethoxazole/trimethoprim 25 μg, tetracycline 30 μg), manufactured by Oxoid, were placed onto the agar plate. The inhibition zone diameter around each antibiotic disk was measured after 5 days of incubation at 15°C.

#### Colony Spreading Motility Testing

The bacterial colony spreading motility testing was performed by spotting 5 μL triplicates of each *F. psychrophilum* isolate from a broth suspension containing 10^9^ CFU mL^–1^ on TYES agar (0.5% agar) plates supplemented with 0.1% baker’s yeast. The colony spreading motility indicated by the colony diameter (mm) of each isolate was evaluated after 6 days of incubation at 15°C.

#### Testing of Proteolytic Activity

The ability of the *F. psychrophilum* isolates to hydrolyze casein, elastin and gelatin was tested on TYES agar (1.5% agar) supplemented with (w/v) skim milk (5%), elastin (0.1%), and gelatin (3%), respectively. A 1 μL volume of a 10^9^ CFU mL^–1^ broth suspension of each isolate was spotted on the agar surface in triplicate, allowed to dry and incubated at 15°C. Following incubation, a positive reaction was indicated by a clear zone in the surrounding turbid agar medium around the inoculum. To estimate the proteolytic activity of each isolate, the clear zone ratio (clearing zone/colony diameter) was determined after 7 (gelatinase) and 10 days (caseinase, elastinase) of incubation.

#### Hemolytic Activity Testing

The hemolytic activity was tested on rainbow trout erythrocytes using a microplate hemolytic assay described by Högfors-Rönnholm and Wiklund (2010a) with some modifications. All *F. psychrophilum* isolates were grown on TYES agar at 15°C for 6 days and suspended in 0.5% NaCl to an OD of 0.45 ± 0.02 at 520 nm, corresponding to a bacterial concentration of approximately 10^8^ CFU mL^–1^. Fish blood was collected by caudal venipuncture of anesthetized rainbow trout and stored in an equal volume of Alsever’s solution. Fresh erythrocytes were harvested by centrifugation (1000 × *g*, 5 min, 4°C), washed with PBS (pH 7.2) and re-suspended to 5% (v/v) in PBS.

Erythrocytes (30 μL) and bacterial suspensions (30 μL) were then mixed and pipetted in triplicates into a round-bottomed microtiter plate and incubated for 24 h at 15°C with 300 rpm agitation. Subsequently, 150 μL of 0.5% NaCl was added to each well, the plate was centrifuged (1000 × *g*, 5 min) and the supernatants were transferred to a flat-bottomed 96-well polystyrene microtiter plate. The absorbance (A) was measured at 540 nm. The negative control (background, A_background_) contained 0.5% NaCl and erythrocytes, and the positive control (total hemolysis, A_100%_) contained hypotonic distilled water for osmotic burst of erythrocytes. Controls were included in triplicates on each plate. The hemolytic activity (HA) was calculated according to: HA = (A-A_background_)/(A_100%_ - A_background_) and the experiment was repeated twice.

#### Adhesion to Polystyrene

Adhesion to polystyrene was examined according to the procedure described by Högfors-Rönnholm et al. (2015). Briefly, the cryopreserved *F. psychrophilum* stocks were cultured for 5 days on TYES agar and recultivated another 3 days before inoculation of bacteria into sterile lake water to an OD_520__nm_ of 1.0, corresponding to approximately 10^9^ CFU mL^–1^. Aliquots of 100 μL were added in triplicate to wells of a flat-bottomed 96-well polystyrene microtiter plate (Nunclon Δ Surface, Nunc) while sterile fresh water was used as a negative control. After static incubation at 15°C for 1 h the contents were discarded and to remove non-adherent cells the wells were washed three times with sterile 0.5% NaCl and air dried. A 125 μL volume of a 0.1% crystal violet (CV) solution was then added to each well and incubated at room temperature for 45 min. After discarding the contents, the plates were washed three times by submersion in a container of tap water and air dried. Then, 150 μL of 96% ethanol was added to each well and incubated at room temperature for 15 min. A 100 μL volume of the solubilized CV was then transferred to a flat-bottomed microtiter plate and the absorbance was quantified in a microplate reader (Victor2, Wallac) at 595 nm. Each isolate was examined in triplicate and the experiment was repeated three times.

#### Estimation of Virulence in Rainbow Trout

The median lethal dose (LD_50_) of each *F. psychrophilum* isolate was estimated in juvenile rainbow trout (mean weight 5 g) from the same cohort obtained from a commercial fish farm. Before start of the virulence experiments, fish were kept under laboratory conditions in tanks with a flow-through of dechlorinated tap water at approximately 13°C with continuous aeration and fed twice a day at 1% of body weight with commercial fish feed. Prior to the challenge trials, fish were fasted for 24 h and anesthetized by immersion in a 0.05 g L^–1^ bath solution of benzocaine just before marking of groups by one of six fin clipping treatments (pectoral fin, pelvic fins, anal fin, adipose fin and dorsal or ventral tail fin) using scissors disinfected with 70% ethanol.

For preparation of *F. psychrophilum* cells for virulence trials, optically adjusted TYES broth suspension containing washed bacterial cells at a concentration of 10^9^ CFU mL^–1^ was used to prepare serial tenfold dilutions (10^8^–10^3^ CFU mL^–1^) of each isolate in 0.5% NaCl. A 50-μL volume of five serial dilutions of *F. psychrophilum* was administered through intramuscular injection into five treatment groups consisting of seven fish each. An equal sized control group was anesthetized, marked and mock-infected with 50 μL of a sterile 0.5% NaCl solution. Disposable 1-mL syringes with a 30-gauge needle were used for the injections. Bacterial viability and concentration of each dilution was verified by colony counting on TYES agar after 7 days of incubation at 15°C. After the injection procedure, each group of fish (*n* = 6), i.e., 42 individuals in total per tested isolate, were transferred to 20 L test aquaria containing aerated and sponge-filtered dechlorinated tap water with a temperature of 13°C.

Mortality was recorded for 21 days during which 80% of the water was renewed daily and its pH and content of ammonia (NH_3_), nitrate (NO_3_), and nitrite (NO_2_) was regularly monitored. Dead and moribund fish were removed as soon as observed. Moribund fish were euthanized by overdose of anesthesia after which the brain was mechanically destroyed. To confirm Koch’s postulate, tissue samples from the kidney and spleen of dead fish were streaked onto TYES agar plates and incubated at 15°C for 7 days. Bacteria forming yellow colonies on the agar plates were identified as *F. psychrophilum* by species-specific PCR as described previously in the text. The LD_50_ for each tested isolate was then estimated by the Reed-Muench method (Reed and Muench, 1938), which requires cumulative mortality to be above 50% in at least one of the treatment groups and below 50% in another.

#### Phage Susceptibility Testing

**FIGURE 1:**
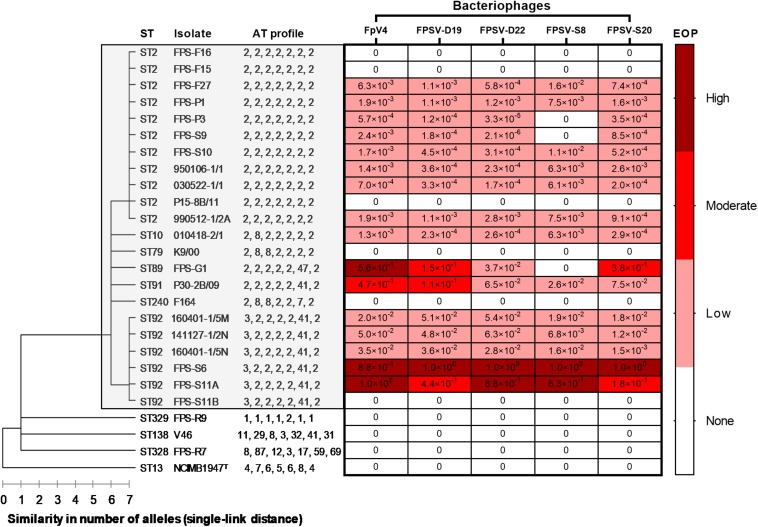
Hierarchical clustering of the allele type (AT) profiles (order: *trpB*, *gyrB*, *dnaK*, *fumC*, *murG*, *tuf*, and *atpA*) using a single-link measure of distance displaying the allelic similarity between the 11 MLST sequence types (STs) identified in this study. Superimposed is the efficiency of plating (EOP) of five bacteriophages on the 26 *F. psychrophilum* isolates included in this study with CC-ST10 genotypes shown within the gray shaded box. The EOP is expressed as high (1–0.5), moderate (0.5 > 0.1), low (0.1 > 10^–7^), or none (0).

As part of the characterization of phenotypic differences between the strains, the phage susceptibility of the bacterial isolates was tested *in vitro* against a selection of 5 phages, representing different host range patterns and different sources, locations and times of isolation (Table 2). These phages included the previously isolated bacteriophage FpV4 (Stenholm et al., 2008) and 4 newly isolated (FPSV-D19, FPSV-D22, FPSV-S8, and FPSV-S20) phages (Table 2 and Figure 1). The new phages were isolated from fish farms during BCWD outbreaks in 2016–2017.

**TABLE 2:** The source of isolation and *F. psychrophilum* hosts used for enrichment and proliferation of the five bacteriophages included in this study.

**Phage**	**Enrichment host**	**Proliferation host**	**Source**	**Country**	**Year of isolation**
FpV4	950106-1/1	950106-1/1	Water sample	Denmark	2005
FPSV-D19	FPS-D10	FPS-S6	Water sample	Denmark	2017
FPSV-D22	FPS-D5	FPS-S6	Fish tissue	Denmark	2017
FPSV-S8	FPS-S2	FPS-S6	Water sample	Sweden	2017
FPSV-S20	FPS-S30	FPS-S6	Water sample	Sweden	2017

Briefly, bacteriophages were isolated from water samples or fish tissue from Danish and Swedish freshwater rainbow trout farms by using single enrichment cultures. The five distinct enrichment hosts for the phages were selected based on shared geographic origin (country) of isolation (Table 2). Approximately 20 mL of a 0.2-μm-filtered water sample was mixed with 2 L 10× TYES broth and with 2 mL of the isolates to be used in the enrichment. Fish were homogenized in a blender and mixed with an equal volume of saline magnesium (SM) buffer (50 mM Tris-HCl, pH 7.5, 99 mM NaCl, 8 mM MgSO_4_) to elute phages. The mixture was centrifuged (5000 × *g*, 15 min, 4°C) and the supernatant 0.2-μm-filtered. One mL of the eluate was mixed with 20 ml of TYES broth and 2 ml of host isolate. The enrichment cultures were incubated for 5–7 days at 15°C with agitation to allow proliferation of lytic *F. psychrophilum* bacteriophages. Following incubation, the culture was 0.2-μm-filtered and the presence of bacteriophages in the filtrate was detected by the double-layer method (Stenholm et al., 2008). For purification of bacteriophages, a single plaque was picked with a plastic Pasteur pipette and the phages were eluted in SM buffer with shaking for 2 h at 15°C or overnight at 4°C. After centrifugation (5000 × *g*, 15 min, 4°C), the supernatant was transferred to a new clean tube and used for re-infection. Bacteriophages were isolated as single plaques by three repeated rounds of plaque purification.

For preparation of high-density bacteriophage stocks, 1 mL bacteriophage stock was added to 20 mL exponentially growing cultures (OD_600__nm_ = 0.2) of a suitable proliferation host (Table 2) and incubated at 15°C for 2–3 days. Lysed bacterial cultures were treated with chloroform and subsequent centrifugation (5000 × *g*, 15 min, 4°C). Phage quantification was performed by small spot assay (Mazzocco et al., 2009). Finally, the efficiency of plating (EOP) of the isolated bacteriophages were determined exposing the *F. psychrophilum* isolates to the same phage titer [∼10^8^ plaque forming units (PFU) mL^–1^] and infectivity was quantified by the small drop plaque assay. PFU were examined after 5 days of incubation at 15°C. Each experiment was performed three independent times and the EOP was presented as the infection efficiency for individual phages, relative to the highest titer obtained for each phage (EOP = 1) on an isolate in the collection.

### Statistical Analyses

As higher LD_50_ values indicate lower virulence, phenotypic characteristics correlating negatively with LD_50_ indicate that when these descriptors get bigger, the LD_50_ value will decrease and the virulence thus increase. Hence, a correlation analysis was performed to identify potential predictors of virulence through identification of statistically significant negative correlations (α < 0.01) between LD_50_ and phenotypic characteristics measured in the study. Statistical significance of the studied phenotypic characteristics on *F. psychrophilum* virulence was tested with a Pearson’s correlations test and a multiple linear regression analysis in GraphPad Prism 8.0.1 with LD_50_ (sample size = 25) as the dependent variable and the measured numerical values for adhesion, colony spreading motility, protease activity (caseinase, gelatinase, and elastinase), hemolytic activity, and antibiotic susceptibility (florfenicol, sulfamethoxazole/trimethoprim, oxolinic acid, and tetracycline) as predictors (*n* = 10). To fulfill the criterion for multiple regression, normality and linearity was verified with a non-significant Shapiro–Wilk test and normal probability plots on residual histogram and P-P plots respectively. The absence of collinearity was also confirmed by the absence of variance inflation factors (VIF) above 3.427 (Table 3). Autocorrelation was checked in IBM SPSS Statistics version 25 with the Durbin Watson test, which was below 2. Homoscedasticity of the data was tested with a Koenker test, and a Breusch–Pagan test using a SPSS macro written by Pryce and Garcia-Granero (2002).

**TABLE 3:** Results of multiple linear regression analysis (*R*^2^ = 0.55) for predictors of *F. psychrophilum* virulence (LD_50_).

**Predictors of LD_50_**	**Estimate**	***SE***	**Lower – upper 95% CI**	**VIF**	**|t|**	***P*-value**
Adhesion	659596	1539817	–2642983 – 3962174	2.618	0.501	0.624
Motility	−18178	22040	−65449 – 29093	2.140	0.428	0.675
Caseinase activity	−912870	594550	−2188054 – 362314	1.778	0.825	0.423
Gelatinase activity	−287295	503758	−1367748 – 793158	1.869	1.535	0.147
Elastinase activity	308817	291965	−317386 – 935021	2.917	0.570	0.578
Hemolytic activity	2568779	1638753	−945997 – 6083555	3.427	1.058	0.308
Florfenicol susceptibility	6303	39398	−78196 – 90803	2.728	1.568	0.139
Oxolinic acid susceptibility	13762	21319	−31963 – 59488	3.042	0.160	0.875
Sulfamethoxazole/trimethoprim susceptibility	35153	51398	−75084 – 145391	1.906	0.646	0.529
Tetracycline susceptibility	18851	17643	−18989 – 56692	1.314	0.684	0.505

*The *P-*value reported in the last column corresponds to the *P-*value of the *t*-test used to check the significance of individual regression coefficients at the 5% level in the multiple linear regression model. LD_50_, median lethal dose; SE, standard error; CI, confidence interval; VIF, variance inflation factors.*

## Results

### Genetic Characteristics and Virulence

Analysis of MLST data of the 26 isolates included in this study together with the 1097 isolates in the global *F. psychrophilum* MLST database^3^ (last access January 17th 2019) showed that the majority of isolates (13/15) obtained from recent BCWD outbreaks (2016–2017) belonged to CC-ST10 and consisted of the genotypes ST2, ST10, ST79, ST89, ST91, and ST92 (Table 1). Two novel STs, ST328 and ST329, not belonging to CC-ST10 were isolated from Russia in 2017. The singleton ST328 did not group into any CCs while ST329 is part of CC-ST1. Of all isolates included in this study, 22 of 26 were of CC-ST10 related STs with ST2 being the most prevalent (*n* = 11) genotype. The genetic serotyping scheme (Rochat et al., 2017) classified 12 *F. psychrophilum* isolates into type 1, 11 isolates into type 2 and 3 isolates into type 0 (Table 1). None of the serotype 0 isolates belonged to CC-ST10.

In the challenge experiments, the examined diseased fish exhibited gross signs commonly associated with BCWD with muscle ulceration and tissue necrosis at the site of injection, and swollen internal organs. Koch’s postulate was fulfilled as *F. psychrophilum* was recovered and identified from the visceral organs (spleen and kidney) of experimentally infected fish. Mortality in fish was generally dose dependent, i.e., with increasing bacterial dose, more mortality was achieved with each *F. psychrophilum* isolate as seen in the survival plots (Supplementary Figure S1). The disease signs were similar for all isolates and the onset of mortality occurred 4–7 days post challenge. None of the mock-infected fish individuals died or showed any symptoms of disease during the challenge trials.

The results from the virulence tests showed a wide range in the degree of pathogenicity in *F. psychrophilum*, with up to 3-log differences in quantifiable LD_50_ between isolates. The LD_50_ of the 26 tested *F. psychrophilum* isolates ranged from 8.9 × 10^3^ (FPS-G1) to 3.1 × 10^6^ CFU (NCIMB1947^*T*^). The *F. psychrophilum* isolates high in virulence were obtained from 6 different countries in the Baltic Sea region and from recent (2016–2017) disease outbreaks. The degree of pathogenicity was determined to correspond to log-differences in LD_50_ and virulence was considered high (*n* = 7) when LD_50_ < 10^5^ CFU, moderate (*n* = 13) when LD_50_ = 10^5^–10^6^ CFU or weak (*n* = 5) when LD_50_ > 10^6^ CFU (Table 1). One *F. psychrophilum* isolate, FPS-S11B, did not induce mortality or necrosis in fish tissue after intramuscular injection of a dose as high as 10^7^ CFU and was thus considered non-virulent and excluded from further statistical analyses (Table 1).

The seven highly virulent *F. psychrophilum* isolates were isolated from recent (2016–2017) disease outbreaks in Denmark, Finland, Germany, Poland, Russia and Sweden. Genetic analyses showed that *F. psychrophilum* isolates high in virulence belonged to the geographically widely distributed clonal complexes CC-ST1 and CC-ST10 (Table 1). Interestingly, the most prevalent *F. psychrophilum* genotype in the global MLST database^4^ ST2 was represented in our study by isolates with high, moderate and weak virulence (Table 1). Moreover, the non-virulent *F. psychrophilum* isolate FPS-S11B and the highly virulent isolates FPS-S6 and 160401-1/5N belonged to the same MLST genotype (ST92), highlighting that pathogenic and non-pathogenic *F. psychrophilum* isolates can share the same ST. All three serotypes caused mortality in rainbow trout, however, all highly virulent *F. psychrophilum* isolates belonged to serotype 1 or 2, while two moderately virulent isolates and the weakly virulent *F. psychrophilum* typestrain NCIMB1947^*T*^ belonged to serotype 0 (Table 1).

### Phenotypic Characteristics and Prediction of Virulence-Associated Traits

**FIGURE 2:**
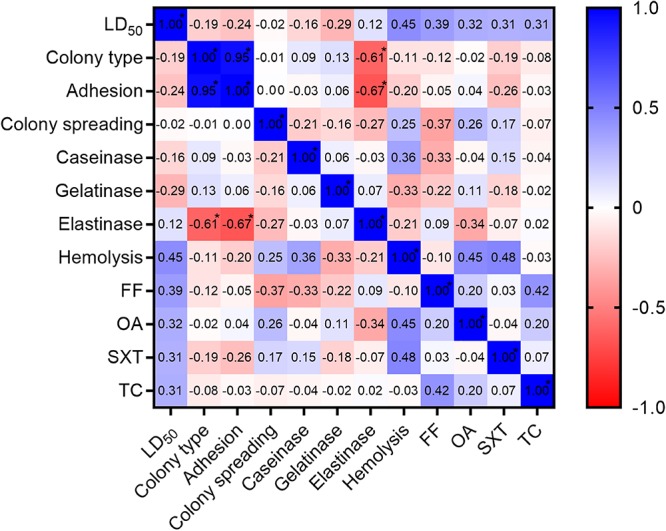
Pearson’s correlation matrix visualized as a heat map plot with LD_50_ and the phenotypic characteristics tested in the study. Asterisk (^*^) indicates significant difference from 0 at the level of α = 0.01. LD_50_, median lethal dose; FF, florfenicol susceptibility; OA, oxolinic acid susceptibility; SXT, sulfamethoxazole/trimethoprim susceptibility; TC, tetracycline susceptibility.

The univariate correlation studies (Figure 2) only showed non-significant associations between the measured phenotypic characteristics and LD_50_. The results from the multivariable analysis (*R*^2^ = 0.55) showed that 55% of the variation in the determined LD_50_ values could be explained by the predictor variables used. The multivariable analysis, however, neither revealed any significant predictors of *F. psychrophilum* virulence (Table 3).

The results from the antibiotic disk diffusion tests showed that all isolates were inhibited by florfenicol and tetracycline, while several of the highly, moderately and weakly virulent isolates were uninhibited by oxolinic acid and sulfamethoxazole/trimethoprim (Table 4). The non-virulent isolate FPS-S11B was deficient in colony spreading motility and proteolytic activity, and also showed the weakest adherence capacity (Table 4). All other isolates showed colony spreading motility and caseinolytic and gelatinolytic activity, while nearly half of the isolates were unable to degrade elastin (Table 4). Interestingly, none of the serotype 0 isolates or highly virulent ST92 genotypes showed any elastinolytic activity, which was present in the three most virulent outbreak isolates. Because elastinase production was observed only in 4 out of 7 highly virulent isolates, it cannot be used as a sole predictor of *F. psychrophilum* virulence. The causation behind the high negative correlation between adhesion and elastinolytic activity (Figure 2) was the inability to degrade elastin observed in the majority (8 out of 10) of the adhesive smooth morphotype isolates. Adhesion was clearly associated with the colony morphology of the isolate, smooth *F. psychrophilum* morphotypes being more adherent than rough (*R*^2^ = 0.90, *P* < 0.0001).

**TABLE 4:** Phenotypic characteristics of 25 virulent *Flavobacterium psychrophilum* isolates and the non-virulent FPS-S11B listed according to decreasing virulence in juvenile rainbow trout (*Oncorhynchus mykiss*).

					**Protease activity (clear zone ratio)**			**Inhibition zone diameter (mm)**			
***F. psychrophilum*** **Isolate code**	**S/R**	**Abs@595 Adhesion**	**Colony spreading (mm)**	**Hemolytic activity**	**Caseinase**	**Gelatinase**	**Elastinase**	**FF**	**OA**	**SXT**	**TC**
FPS-G1	S	0.355 ± 0.014	34.3 ± 1.3	0.345 ± 0.025	3.6 ± 0.1	5.2 ± 0.4	3.3 ± 0.2	43	8	0	41
FPS-F15	R	0.158 ± 0.004	43.0 ± 1.2	0.202 ± 0.055	3.3 ± 0.1	4.5 ± 0.1	2.6 ± 0.2	47	0	0	58
FPS-P1	R	0.153 ± 0.005	27.0 ± 1.2	0.093 ± 0.022	2.5 ± 0.1	5.1 ± 0.3	2.8 ± 0.1	66	0	9	41
FPS-R9	S	0.552 ± 0.012	32.0 ± 6.5	0.163 ± 0.029	2.9 ± 0.1	6.1 ± 0.1	1.0 ± 0.0	54	43	0	70
FPS-P3	R	0.155 ± 0.006	44.0 ± 5.0	0.096 ± 0.007	2.6 ± 0.1	5.2 ± 0.4	2.7 ± 0.3	36	0	0	30
FPS-S6	S	0.598 ± 0.014	28.7 ± 1.3	0.250 ± 0.047	3.2 ± 0.1	4.4 ± 0.3	1.0 ± 0.0	56	0	0	49
160401-1/5N	S	0.415 ± 0.008	28.3 ± 2.0	0.358 ± 0.012	3.7 ± 0.1	4.7 ± 0.4	1.0 ± 0.0	50	0	7	44
P30-2B/09	S	0.568 ± 0.017	22.7 ± 1.9	0.144 ± 0.004	3.1 ± 0.1	4.7 ± 0.3	1.0 ± 0.0	44	0	0	37
F164	R	0.134 ± 0.003	10.3 ± 0.7	0.087 ± 0.029	3.3 ± 0.1	4.7 ± 0.2	2.4 ± 0.1	56	9	0	58
FPS-F16	R	0.142 ± 0.004	37.3 ± 0.9	0.194 ± 0.066	3.2 ± 0.1	4.7 ± 0.1	2.8 ± 0.1	54	7	0	60
V46	R	0.138 ± 0.003	38.7 ± 3.0	0.829 ± 0.077	4.2 ± 0.3	5.0 ± 0.3	1.0 ± 0.0	40	31	12	44
FPS-S11A	S	0.515 ± 0.011	45.0 ± 2.1	0.061 ± 0.012	3.3 ± 0.2	4.7 ± 0.3	1.0 ± 0.0	52	6	9	64
990512-1/2A	R	0.141 ± 0.004	19.3 ± 3.0	0.318 ± 0.036	3.4 ± 0.1	5.0 ± 0.3	3.7 ± 0.1	49	0	8	55
141127-1/2N	S	0.452 ± 0.008	26.3 ± 2.4	0.143 ± 0.019	3.3 ± 0.1	5.2 ± 0.1	1.0 ± 0.0	50	8	7	45
P15-8B/11	R	0.162 ± 0.005	24.0 ± 1.0	0.188 ± 0.029	3.1 ± 0.1	4.9 ± 0.1	3.1 ± 0.1	57	6	0	58
160401-1/5M	S	0.365 ± 0.015	20.0 ± 2.6	0.321 ± 0.085	3.6 ± 0.1	5.8 ± 0.4	1.0 ± 0.0	49	7	0	44
FPS-F27	R	0.132 ± 0.004	38.7 ± 1.9	0.183 ± 0.016	3.1 ± 0.1	5.7 ± 0.3	2.7 ± 0.2	44	0	8	48
FPS-R7	S	0.526 ± 0.027	24.3 ± 1.5	0.257 ± 0.048	2.8 ± 0.2	4.9 ± 0.1	1.0 ± 0.0	57	16	0	67
FPS-S9	R	0.138 ± 0.004	28.0 ± 1.0	0.100 ± 0.001	3.6 ± 0.2	5.6 ± 0.6	2.9 ± 0.2	52	10	0	64
030522-1/1	R	0.127 ± 0.004	15.0 ± 1.5	0.344 ± 0.052	3.4 ± 0.1	4.7 ± 0.2	3.0 ± 0.1	59	0	8	53
FPS-S10	R	0.137 ± 0.005	15.3 ± 1.8	0.127 ± 0.009	3.3 ± 0.1	5.3 ± 0.3	2.8 ± 0.1	54	0	7	65
010418-2/1	R	0.135 ± 0.005	13.0 ± 1.1	0.357 ± 0.007	3.5 ± 0.1	4.8 ± 0.3	3.0 ± 0.1	54	0	7	64
K9/00	R	0.177 ± 0.004	25.0 ± 2.1	0.381 ± 0.001	3.2 ± 0.1	4.8 ± 0.4	2.7 ± 0.1	64	44	0	44
950106-1/1	S	0.397 ± 0.011	24.7 ± 0.3	0.359 ± 0.067	3.2 ± 0.1	4.8 ± 0.3	2.9 ± 0.1	54	0	7	59
NCIMB1947^*T*^	R	0.117 ± 0.002	54.7 ± 0.3	0.746 ± 0.216	2.7 ± 0.1	4.1 ± 0.1	1.0 ± 0.0	56	32	12	65
FPS-S11B	R	0.110 ± 0.003	7.7 ± 0.3	0.222 ± 0.022	1.0 ± 0.0	1.0 ± 0.0	1.0 ± 0.0	57	8	0	62

*R indicates rough colony morphology and S indicates smooth. A clear zone ratio of 1.0 ± 0.0 indicates no activity. FF, florfenicol; OA, oxolinic acid; SXT, sulfamethoxazole/trimethoprim; TC, tetracycline.*

### Phage Susceptibility

In general, 16 out of 26 *F. psychrophilum* strains were sensitive to at least three bacteriophages, with a 10^6^-fold variation in EOP for each phage depending on the host (Figure 1). Only MLST genotypes belonging to the CC-ST10 cluster were infected, whereas more distantly related strains were resistant to infection by the five phages. However, within CC-ST10, there was no direct correlation between ST and phage susceptibility, and each phage was able to infect strains from 4 to 5 different STs. The group of phage-sensitive *F. psychrophilum* isolates included 5 (FPS-G1, FPS-P1, FPS-P3, FPS-S6, and 160401-1/5N) of the seven most virulent strains which represented three different STs (ST2, ST89, and ST92) within CC-ST10, whereas the other highly virulent strains (FPS-F15 and FPS-R9) were resistant to phage infection (Figure 1).

## Discussion

Pathogenic diversity within bacterial species is a known phenomenon and the study of virulence in bacteria causing infectious diseases in fish has primarily been focused on studying one or a few characteristics at a time (Sudheesh et al., 2012). In many bacterial species, the determinants regulating virulence are however difficult to differentiate because pathogenic strains can express several virulence factors simultaneously and a variety of factors can contribute to the overall pathogenicity (Cutler, 1991). Although virulence is considered a universal pathogen characteristic, it is expressed only in a susceptible host. Therefore, a measure of virulence involves an assessment of a host-pathogen interaction, most commonly morbidity or mortality. Identification of factors required for virulence is not only important for understanding the development of disease, but also for discovery of targets for pathogen control. Alternatives to antibiotic administration that kill or inhibit growth of pathogens could be achieved by development of anti-virulence drugs that disrupt or inhibit key pathogenicity or virulence factors that are general across the species including different strains.

Here we used a large collection of genetically and phenotypically characterized *F. psychrophilum* isolates collected from BCWD outbreaks in the Baltic Sea region for exploration of predictors for pathogenicity and virulence as measured by mortality (LD_50_) in juvenile rainbow trout through a reproducible intramuscular injection-based infection model. Inclusion of the weakly virulent reference strain NCIMB1947^T^ and a large number of isolates demonstrating a wide 3-log range in LD_50_ enabled us to evaluate the relationships between specific genetic and phenotypic factors and virulence.

In a previous virulence study, Garcia et al. (2000) showed a 2-log variation in LD_50_ among six outbreak isolates of *F. psychrophilum* after intramuscular injection experiments in juvenile rainbow trout. The observed wide range in virulence among *F. psychrophilum* strains in previous and this study sets limits when selecting for the range of test doses (CFU) for LD_50_ determination, which in turn affects the accuracy of LD_50_ predictions. Some variation in the predicted LD_50_, particularly among isolates of the same genotype, might be explained by experimental variability that may have been further revealed in experimental replicates. However, for LD_50_ determination of a total of 26 isolates in this study (Table 1), we used five test doses with a separation factor of 10 (Supplementary Figure S1), and 7 fish individuals for each dose without replication to minimize the number of experimental animals to be sacrificed, while still meeting the minimum standards described in the Organisation for Economic Cooperation and Development [OECD] (2019) Guidelines for Testing of Chemicals (Test No. 203: Fish, Acute Toxicity Test). For a more definitive test of LD_50_, smaller separation factors and replication are likely necessary.

Due to the lack of predictors of pathogenicity in *F. psychrophilum*, there was no rationale for *a priori* exclusion or prioritization of potential virulence factors in our multivariable analysis (McClelland et al., 2006). Instead of estimating the relative contributions of individual virulence factors, our aim was to identify large effects of selected phenotypic characteristics using the largest number of *F. psychrophilum* isolates tested for *in vivo* virulence to date.

### Genetic Predictors of Virulence

The resolution of the current MLST scheme was not sufficient to differentiate highly virulent from weakly or non-virulent *F. psychrophilum* isolates. Interestingly, pathogenic CC-ST10 strains showed up to a 100-fold variation in virulence not only within the complex but also within the most prevalently isolated ST2. The observed variation in virulence within isolates of identical STs could be explained by diversity in virulence gene content or by differences in the expression of genes. The identification of weakly virulent outbreak isolates of CC-ST10 indicates that the severity of BCWD outbreaks is attributed not only to the virulence of *F. psychrophilum*, but also to the state of host fish as well as environmental factors.

With the recently developed PCR-based serotyping scheme, Rochat et al. (2017) showed that the genetically determined serotypes 0, 1 and 2 correspond to the previously proposed *F. psychrophilum* serogroups FpT, Th and Fd, respectively, determined by slide-agglutination tests (Lorenzen and Olesen, 1997). Serotype 1 (serogroup Th) and 2 (serogroup Fd) have previously been shown to be predominant in BCWD outbreaks (Lorenzen and Olesen, 1997; Dalsgaard and Madsen, 2000) and more virulent than serotype 0 (serogroup FpT) in experimentally infected rainbow trout (Madsen and Dalsgaard, 2000; Madetoja et al., 2002). A significant association between the serotype and the host fish species has also been found, with serotype 1 and 2 being more prevalent in rainbow trout and strains infecting coho salmon belonging more often to serotype 0 (Rochat et al., 2017). These findings are in keeping with the results from the present study where the ten most virulent outbreak isolates belonged to serotype 1 and 2 and none of the serotype 0 isolates belonged to the dominant rainbow trout-associated (Nilsen et al., 2014) clonal complex CC-ST10 (Table 1). The serotype diversity (serotype 1 and 2) found among moderately virulent ST2 isolates (Table 1) might also explain some of the observed variation in ST2 virulence and further complicate control of BCWD by vaccination.

### Phenotypic Predictors of Virulence

CC-ST10 isolates from BCWD outbreaks in rainbow trout have previously been associated with a higher ability to adhere to inert and mucus coated surfaces compared to genetically distinct *F. psychrophilum* strains isolated from other sources (Sundell and Wiklund, 2015). Our results confirmed previous observations that smooth colony phenotypes are superior to rough in adhering to inert surfaces (Högfors-Rönnholm et al., 2015) but equally virulent (Figure 2; *R*^2^ = 0.04, *P* = 0.13) when injected into rainbow trout (Högfors-Rönnholm and Wiklund, 2010b). Similarly, virulence in *F. psychrophilum* could not be predicted by the hemolytic activity in the microplate assay, but here, contrary to previous findings by Högfors-Rönnholm and Wiklund (2010a), we did not detect any significant differences (*R*^2^ = 0.01, *P* = 0.59) in the activity between rough and smooth isolates.

Gliding motility facilitates the movement of bacteria along surfaces in *Flavobacterium* species and results in the spreading of colonies. Gliding motility in *F. psychrophilum* is not only important for colonization, but also for expression of virulence factors through cooperative function with secretion systems (Pérez-Pascual et al., 2017). Gliding motility in *F. psychrophilum* is associated with the protein secretion system PorSS which is also believed to influence secretion of extracellular enzymes (Castillo et al., 2015), suggesting that these phenotypic properties are involved in virulence. Studies with *F. psychrophilum* mutants deficient in colony spreading showed a significant reduction in gelatinolytic and caseinolytic activity, and attenuated virulence compared to the wild-type isolate (Pérez-Pascual et al., 2017). Excretion of proteases degrading casein and gelatin is a common characteristic of *F. psychrophilum* whilst elastinase production varies between strains (Bertolini et al., 1994, Sundell and Wiklund, 2015). The unique genetic determinant allowing for elastin degradation was just recently discovered (Rochat et al., 2019) and a strong elastinolytic activity was furthermore associated with CC-ST10 genotypes. Although elastinase production was required for maximal virulence in our infection model, elastinolytic activity was not a significant predictor of virulence. The absence of elastinolytic activity in two highly virulent outbreak isolates of genotype ST92 belonging to CC-ST10 indicates that elastinase production is neither obligatory for high virulence nor pathogenicity in rainbow trout. Proteases secreted by fish pathogens have the potential to destroy proteins, which constitute the structure and function of the host fish tissues and proteins involved in host immune defense. However, demonstrating that such interactions occur during BCWD outbreaks in fish farms is difficult as expression of proteases in *F. psychrophilum* may be under complex regulation.

Our results demonstrated a high variability in caseinolytic and gelatinolytic activity, and colony spreading within the groups of high, moderate and weak virulence, and that the inability of one isolate (FPS-S11B) to cause mortality in fish was associated with its unique lack of protease production and deficiency in colony spreading (Table 4). Our findings are supported by results obtained through transposon-based mutagenesis of *F. psychrophilum*, where inhibition of colony spreading and extracellular proteolytic activity resulted in a complete loss of pathogenicity in rainbow trout (Pérez-Pascual et al., 2015). We therefore conclude that some levels of colony spreading motility and protease production are likely to be required for pathogenicity in *F. psychrophilum*, but that no further gains in virulence will necessarily occur if colony spreading and/or proteolytic activity increase.

### Antibiotic Resistance and Virulence

Recent in depth analysis of genome data revealed that recombination is the key driver of *F. psychrophilum* evolution and genes specific to CC-ST10 suggests a possible link between antibiotic resistance and virulence (Duchaud et al., 2018). Virulence and antibiotic resistance are similar in that some of the determinants are transmitted between species by horizontal gene transfer. Additional factors that are common to virulence and resistance include important adaptation-related processes that activate or repress the expression of various genes (Yeung et al., 2011). Although antibiotic resistance is not in itself a virulence factor, the relationship between resistance and virulence can be of benefit to a pathogen in environments such as fish farms where antibiotics are frequently used. Therefore, antibiotic resistance may be considered a virulence-like factor, which antibiotic-resistant pathogens or strains can utilize in environments where selective antibiotic pressure prevails (Beceiro et al., 2013).

Perhaps alarmingly, a recent temporal genetic analysis of *F. psychrophilum* isolates from BCWD outbreaks in Swedish fish farms between 1988 and 2016 revealed a shift in domination from quinolone-sensitive ST79 clones in 1988–1997 to resistant ST92 clones in 2011–2016 (Söderlund et al., 2018). Quinolone-resistance in *F. psychrophilum* has also recently been associated with severe BCWD outbreaks in Finland (Sundell et al., 2013) and Norway (Shah et al., 2012). Although we did not find any associations between decreasing antibiotic inhibition zone diameters and increasing virulence, our study showed that the CC-ST10 isolates FPS-S6, 160401-1/5N, FPS-S11A, 141127-1/2N, and 160401-1/5M of ST92 isolated between 2014 and 2017 were less inhibited by oxolinic acid in the disk diffusion tests and more virulent compared to the CC-ST10 isolate K9/00 of ST79 isolated in year 2000 (Table 4). These findings provide additional support to the hypothesis of a more recent dissemination of the virulent quinolone-resistant *F. psychrophilum* clone ST92 to the Baltic Sea region (Söderlund et al., 2018).

According to the global MLST database^5^, ST92 was first reported in Denmark in 2006 and thereafter in several countries including Finland, France, Sweden and Switzerland indicating a rapid and wide geographical dissemination of a quinolone-resistant pathogenic clone. Perhaps even more alarmingly, one highly virulent outbreak strain (FPS-P3) reportedly responsible for massive fish farm mortality in 2017 belonged to the most prevalent *F. psychrophilum* genotype ST2 of CC-ST10 and showed the least inhibition by florfenicol and tetracycline (Table 4), both commonly used antibiotics for treatment of BCWD. Reduced antibiotic susceptibility in *F. psychrophilum* isolated from disease outbreaks is however not restricted to the Baltic Sea region as it has also frequently been observed among North and South American isolates (Hesami et al., 2010; Miranda et al., 2016). The development of resistance in highly pathogenic and prevalent *F. psychrophilum* CC-ST10 variants and their potential of rapid international spread emphasizes that there is an urgent need for an alternative therapeutic agent to antibiotics for control of BCWD.

### Phage-Susceptibility of CC-ST10

The isolation and identification of lytic bacteriophages able to infect prevalent and particularly antibiotic-resistant *F. psychrophilum* variants could be of crucial importance for the development of a novel control intervention for BCWD. Interestingly, the isolated bacteriophages showed lytic activity against most highly virulent outbreak isolates and, particularly importantly, against several important CC-ST10 genotypes including the emerging quinolone-resistant clone ST92 (Söderlund et al., 2018). The phage-susceptible strains belonged to distinct STs and represented isolates from 5 different countries, isolated over a period of two decades. This emphasizes the broad host-range and long-term stability of the infectious properties of individual *F. psychrophilum* phages, providing further reason for investigating the potential of phage-based control of BCWD. However, biosafety issues and the emergence of phage-resistance in the bacterial population with still unpredictable functional implications in *F. psychrophilum* need thorough investigation for bacteriophages to be considered in applications to control BCWD in fish farm environments.

## Conclusion

We have characterized the *in vivo* virulence of a large collection of *F. psychrophilum* isolates obtained from BCWD outbreaks in the Baltic Sea region, and correlated this with the expression of several phenotypic virulence-related characteristics *in vitro*. Our results show that isolates of *F. psychrophilum* retrieved from BCWD outbreaks can show up to a 3-log variation in virulence, but exhibit similar genotypic and phenotypic characteristics. However, colony spreading motility and production of caseinolytic and gelatinolytic enzymes were universal observable characteristics amongst isolates pathogenic to rainbow trout, and may function as *a priori* plausible predictors of *F. psychrophilum* pathogenicity. This collection of *F. psychrophilum* isolates represents a valuable resource for future research on virulence-related factors through comparative genomic analyses and gene expression studies, and the collection of lytic phages with the ability to infect and kill highly virulent antibiotic-resistant variants provides a necessary tool kit for continued research aimed at estimating the potential of phage-based control of BCWD.

## Nucleotide Sequence Accession Numbers

Nucleotide sequences have been deposited in the GenBank database under accession numbers MK776535–MK776667.

## Data Availability

The datasets generated for this study can be found in GenBank and Supplementary Figure S1, MK776535–MK776667.

## Ethics Statement

All animal experiments were performed in Finland under project (ESAVI/4225/04.10.07/2017) and personal license issued by the Animal Experimental Board (Eläinkoelautakunta, ELLA).

## Author Contributions

KS: animal experimentation, data preparation and interpretation, and writing the bulk of the manuscript. LL: bacterial isolation, DNA extraction, PCR and phenotypic characterization. PN: MLST data analysis, gene sequence submission and participation in data interpretation. JJ and DC: bacterial and phage isolation, phage-susceptibility testing and data preparation. ID, VD, and LM: bacterial isolation, hemolytic activity testing and data preparation. MM: intellectual contribution throughout the study, interpretation of data, manuscript preparation, and acquisition of funding. TW: intellectual contribution throughout the study, interpretation of data, and manuscript preparation. All authors read and approved the final manuscript.

## Conflict of Interest Statement

The authors declare that the research was conducted in the absence of any commercial or financial relationships that could be construed as a potential conflict of interest.

## Footnotes

1 http://pubmlst.org/fpsychrophilum

2 http://eburst.mlst.net

3 http://pubmlst.org/fpsychrophilum

4 http://pubmlst.org/fpsychrophilum

5 http://pubmlst.org/fpsychrophilum

## Supplementary Material

FIGURE S1 Survival plots after intramuscular challenge with 25 *F. psychrophilum* isolates determined for the median lethal dose (LD_50_) value in juvenile rainbow trout (*Oncorhynchus mykiss*). Each isolate was injected in five serial 10-fold doses (CFU) into 7 fish per each dose. Control fish were mock-infected with 0.5% NaCl. The isolates were categorized into highly (LD_50_ < 10^5^), moderately (LD_50_ = 10^5^–10^6^), or weakly virulent (LD_50_ > 10^6^) based on the determined LD_50_ value. The degree of virulence is enclosed in parentheses after the isolate code on each plot.
