# Supplementary figures and images for: Phenotypic and Genetic Predictors of Pathogenicity and Virulence in Flavobacterium psychrophilum
Source: Front Microbiol. 2019 Jul 24;10:1711. doi: 10.3389/fmicb.2019.01711 (PMC6668605; doi:10.3389/fmicb.2019.01711)

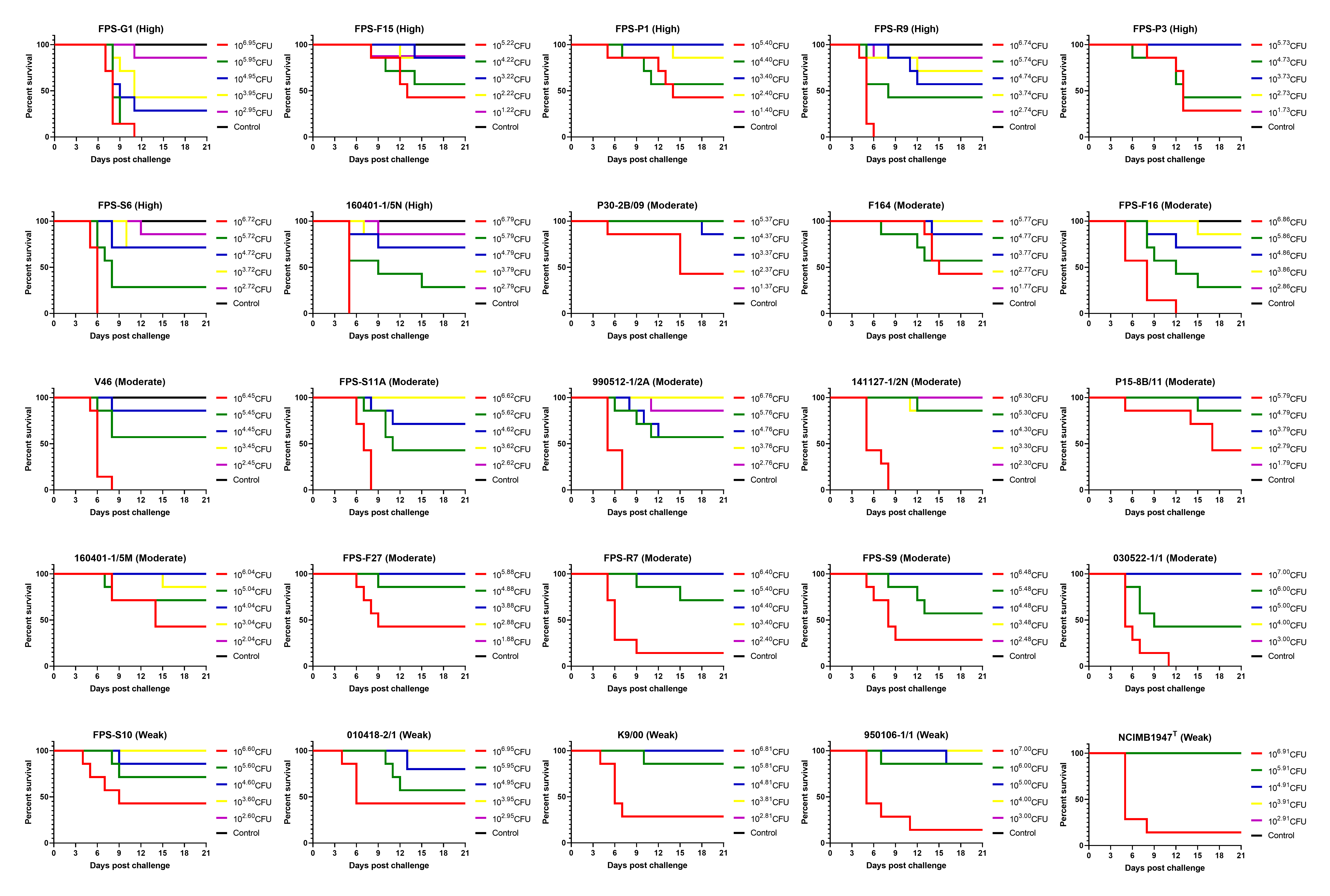

Supplement: FIGURE S1 — Survival plots after intramuscular challenge with 25 F. psychrophilum isolates determined for the median lethal dose (LD50) value in juvenile rainbow trout (Oncorhynchus mykiss). Each isolate was injected in five serial 10-fold doses (CFU) into 7 fish per each dose. Control fish were mock-infected with 0.5% NaCl. The isolates were categorized into highly (LD50 < 105), moderately (LD50 = 105–106), or weakly virulent (LD50 > 106) based on the determined LD50 value. The degree of virulence is enclosed in parentheses after the isolate code on each plot. [file Image_1.TIF]
